# Notes and Comments

**Published:** 1899-10

**Authors:** 


### Notes and Comments.[1]

OFFICE OF THE STATE SOCIETIES.—In an admirable editorial in the August *Dental Brief,* Dr. Litch says,—

"If the cause of dental education is to be upheld and advanced; if the laws regulating dental practice are to be enforced; if ethical standards are to be maintained, it is chiefly through the State society that it must be done. By every reputable practitioner be it remembered that to do these things the society needs membership; it needs personal work and personal influence; it needs YOU."

NOVEL METHOD OF FILLING TEETH.—Dr. Carl Jung, Director of the Dental Institute at the University of Heidelberg, has given food for reflection to dentists in search of a new filling for teeth in which the crowns have been destroyed, leaving the pulp intact. He suggests tin as a material suitable in every respect. His method, given in *The Dentist,* is as follows: "After the cavity has been prepared in the usual manner, and the edges thoroughly finished, the overlapping parts are filled with a little cement or plaster, after which an impression is taken with a little piece of modelling composition, and a plaster model is cast which is thoroughly dried. A little tin is now introduced into the cavity reproduced in the plaster; flow it (use no soldering solution) with the hot soldering iron and press with a piece of leather or linen, as in preparing a flat tooth with tin back. Filed and finished, the tin filling itself is now complete. With some practice it is easy to fill even complicated cavities with the soldering iron, without the tin overflowing the sides. If the soldering iron is not too hot, it is possible to bring the tin into a state in which it does not flow directly, but can be worked with the spatula like wax. A matrix of mouldine or the like could be built around it. Dr. Jung speaks very highly of the results he has achieved.

[1] The assistant editor solicits contributions for this department,—new methods, new remedies and formulas, or any short practical note which may prove of value to the practitioner or student. Address 1338 Walnut Street, Philadelphia.

Carbonized Cotton as a Root-Canal Filling.—In an interesting paper on the art of filling teeth, published in *The Dentist*, the following method of filling root-canals, as practised by Dr. Elof Förberg, of Stockholm, is given. The usual materials, Dr. Förberg claims, do not meet the requirements, for while gold may be made aseptic it is impossible to introduce it into curved and flattened canals; wood and gutta-percha are not aseptic, and decompose. Cements are irritating, and soon dissolve. Speaking of his own method, he says,—

"After years of experimentations to modify cotton in a way to make it insoluble and non-decomposing, I finally obtained in the *carbonized cotton* the material which I was searching for. Carbonized cotton differs in many respects from the hitherto known modifications of carbon. It seems to be a modification between the diamond and the graphite. Like the former, it is a non-conductor of electricity and heat; like the latter, it hardly burns. Like the charcoal, it is a good absorber of gaseous bodies, and excels it on account of its higher porosity. This porous, soft, and flexible carbon, by itself a disinfectant, is also an excellent antiseptic, owing to the addition of anhydrous boracic acid, with which every fibre of it is impregnated. The carbonized cotton was used by me first in the filling of root-canals. All difficulties arising on account of its brittleness and black color were reduced to a minimum after a short time, so that I can say it is excellent for various purposes. The property of carbon to absorb gases and liquids is of importance in the filling of root-canals. All septic masses which may appear in spite of careful treatment are readily taken up by it and made harmless. Carbonized cotton is entirely aseptic, and can be brought to a red heat before use. It may be introduced in the canals as such, or combined with any good antiseptic. No irritation ever results within the tooth nor on the surrounding parts."

